# Trends in Drug and Polysubstance-Related Deaths in the State of Texas: County Comparisons

**DOI:** 10.7759/cureus.79175

**Published:** 2025-02-17

**Authors:** Ernst J Nicanord, Wei-Chen (Miso) Lee, Sarah A Konopnicki, Kendall Campbell

**Affiliations:** 1 Family Medicine, University of Texas Medical Branch at Galveston, Galveston, USA

**Keywords:** cdc opioid guidelines, drug-related deaths, opioid misuse, synthetic fentanyl, texas counties

## Abstract

Background: The opioid epidemic remains a significant public health challenge, with a substantial number of overdose deaths occurring annually. Synthetic opioids, particularly illicitly manufactured fentanyl, account for a significant portion of these fatalities, with a notable increase in adolescent deaths. In Texas, despite a decrease in opioid prescriptions, overdose deaths from both legal and illegal opioids have increased. The state’s current overdose tracking program lacks comprehensive reporting, suggesting the crisis may be worse than reported due to incomplete data from rural counties affected by border crossings. This paper examines the pattern of opioid overdose deaths in Texas over the last seven years and postulates that the extent of the opioid crisis in Texas may be worse than what is officially reported due to incomplete data from rural counties.

Methods: This study presents a trend analysis based on the Texas Department of State Health Services’ surveillance data from 2016 to 2022. The analysis highlights trends in total polysubstance and drug-related deaths, the number of affected counties, and the average number of deaths per county. Additionally, the study identifies counties with increasing death rates over the seven-year period.

Results: The analysis revealed a consistent increase in drug-related and polysubstance-related deaths from 2016 to 2022, along with a rise in the number of counties reporting these deaths. Additionally, a statistically significant association was found between the year and the number of deaths. The data indicates the significant impact of the COVID-19 pandemic, as evidenced by a dramatic surge in polysubstance-related deaths from 2019 to 2020.

Conclusions: The opioid crisis in Texas calls for a comprehensive strategy that includes improved data collection, equitable resource distribution, and evidence-based legislative reforms. This approach, which should be guided by empirical data, would not only address the immediate crisis but would also contribute to the broader discourse on public health policy and practice.

## Introduction

States and local public health authorities continue to struggle with the growing challenge of substance overdose. The Centers for Disease Control and Prevention (CDC) reported over 100,000 overdose deaths nationwide between May 2020 and April 2021. Of particular concern is the alarming prominence of synthetic opioids, notably illicitly manufactured fentanyl, that account for 64.0% of these fatalities [1] and the corresponding increase of overdose deaths in adolescents due to illicitly manufactured fentanyl [2].

Despite the nationwide surge in opioid overdose death rates, southern states, notably Texas, have remained relatively underrepresented in comprehensive studies of overdose patterns [3]. Texas is the second largest state by population in the United States and makes up nearly half of the entire U.S-Mexico border, which has been associated with drug tourism, drug-related violence, and a major source of fentanyl [4]. In Texas, the Department of State Health Services reported an increase in more than 75% of the state’s drug overdose deaths in the past five years [5].

A study on opioid prescribing behavior and overdose fatalities in Texas between 2013 and 2017 showed that, while opioid prescriptions have decreased, overdose deaths from both legal and illegal opioids increased [6]. Overdose fatalities continued to uptrend during the COVID-19 pandemic, which exacerbated the challenges already present in the ongoing opioid epidemic. Drug overdose-related deaths increased in the United States both before and after the onset of COVID-19, while in Canada, such deaths stabilized before the pandemic but rose afterward [7]. Furthermore, opioid-related emergency medicine utilization rose significantly at the height of the COVID-19 pandemic [8]. In fact, in the early months of the pandemic, drug overdose ranked third among the most common causes of death in Black, Hispanic, and white Texans aged 25 to 44 [9].

Nevertheless, the state’s current program aimed at tracking and preventing drug overdose still lacks a robust and comprehensive system for reporting opioid overdoses that encompasses all counties within Texas. This paper examines the pattern of opioid overdose deaths in Texas over the last seven years and postulates that the extent of the opioid crisis in Texas may be worse than what is officially reported due to incomplete data from rural counties.

## Materials and methods

Data source

The data for this study was drawn from Texas Health Data-Opioids, the drug-related and polysubstance-related deaths surveillance system administered by the Texas Department of State Health Services. Data were collected for the years 2016-2022 [10]. Texas counties report data to the state-level statistics center so that the state government can track prevention efforts and provide needed support to ensure public safety.

Drug-related deaths are defined as deaths caused by drug use based on a person’s death certificate [5]. The causes of death were identified through the ICD-10 codes, including X40-X44, X60-X64, X85, and Y10-Y14. Next, polysubstance-related deaths are defined by ICD-10 codes including Opioids (T40.0 Opium, T40.1 Heroin, T40.2 Other opioids, T40.3 Methadone, T40.4 Other synthetic narcotics, and T40.6 Other and Unspecified Narcotics), Psychostimulants (T40.5 Cocaine, T42.4 Benzodiazepine, T40.7 Cannabis, and T43.6 Psychostimulants), and Psychotropics (T43). County data is presented based on the county of residence or county of occurrence, whether the person is a Texas resident or not. Counts of 1-9 are suppressed to protect privacy, while counts of 1-20 are suppressed for reliability [11]. It is not possible to determine whether an opioid was illegally produced or pharmaceutical or how an opioid was obtained. Counties that did not have data indicate no drug-related deaths in a certain year. The study does not require IRB approval, given it does not involve human subjects or Protected Health Information (PHI).

Variables

The first outcome is the number of drug-related deaths in Texas by county from 2016 to 2022, the second outcome is the number of polysubstance-related deaths in Texas by county from 2016 to 2022, and the third outcome is the number of Texas counties with drug-related or polysubstance-related deaths. We further generated two new variables to get the average number of deaths per county. The independent variables are year and county.

Data analysis

A table was used to present the trend of the total number of deaths, total number of counties, and average number of deaths per county. Charts were used to present the trend of the average number of deaths per county on a map. The association between year and number was examined using t-test analysis, and p-values less than 0.05 were considered statistically significant. All analyses were done by the Stata v17 statistical software program.

## Results

Table 1 provides the description of the total number and average of deaths per year and per cause. The number of counties with drug-related deaths increased from 41 to 58, and the number of counties with polysubstance-related deaths also increased from 13 to 24 over seven years. Both the total number and average number are also steadily increasing. In 2016, there were 2,842 drug-related deaths and 629 polysubstance-related deaths, compared to 5,305 drug-related deaths and 1,921 polysubstance-related deaths by 2022. Additionally, there is a dramatic change in the number of polysubstance-related deaths from 2019 to 2020, implying that the beginning of the COVID-19 pandemic had a great impact on the health of Texans.

**Table 1:** Total Drug-Related and Polysubstance-Related Deaths by Year *Polysubstance-related deaths: Opioids (T40.0 Opium, T40.1 Heroin, T40.2 Other opioids, T40.3 Methadone, T40.4 Other synthetic narcotics, T40.6 Other and Unspecified Narcotics) + (T40.5 Cocaine or T42.4 Benzodiazepine or T40.7 Cannabis or T43.6 Psychostimulants)

Year	2016	2017	2018	2019	2020	2021	2022	p-value
All Drugs								0.283
Total number of deaths	2,842	2,946	2,936	3,132	4,110	4,832	5,305	
Total number of counties	41	46	39	44	53	53	58	
Average # of deaths	59.88	55.78	63.92	61.25	70.06	83.26	83.21	
Polysubstance Only*								0.012
Total number of deaths	629	751	720	851	1,251	1,697	1,921	
Total number of counties	13	14	13	13	17	22	24	
Average # of deaths	39.77	42.14	49.54	61.41	67.00	68.67		

**Figure 1:**
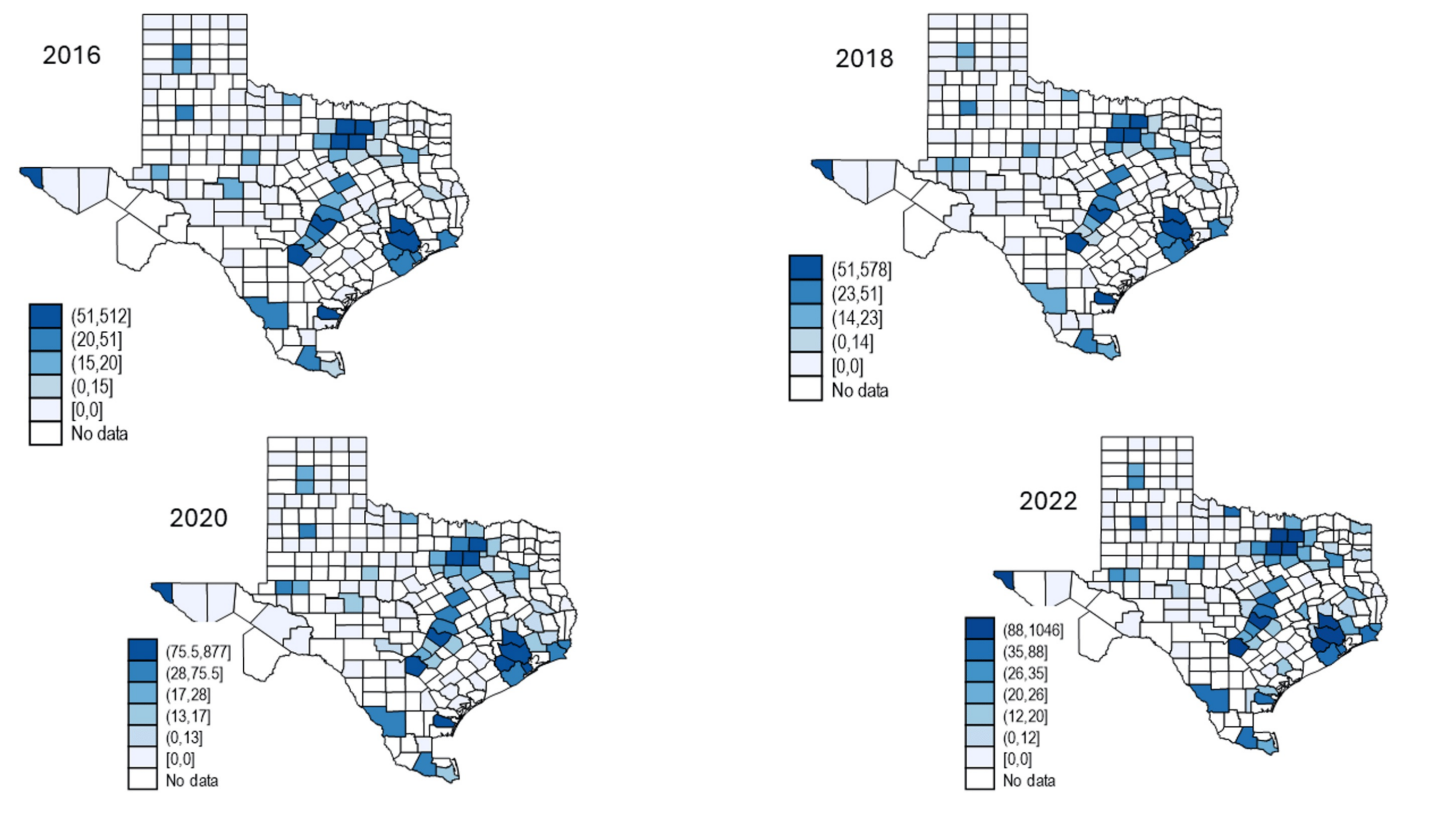
Drug-Related Deaths by County and Year The image was created by the authors of this article using the STATA v17 software program.

**Figure 2:**
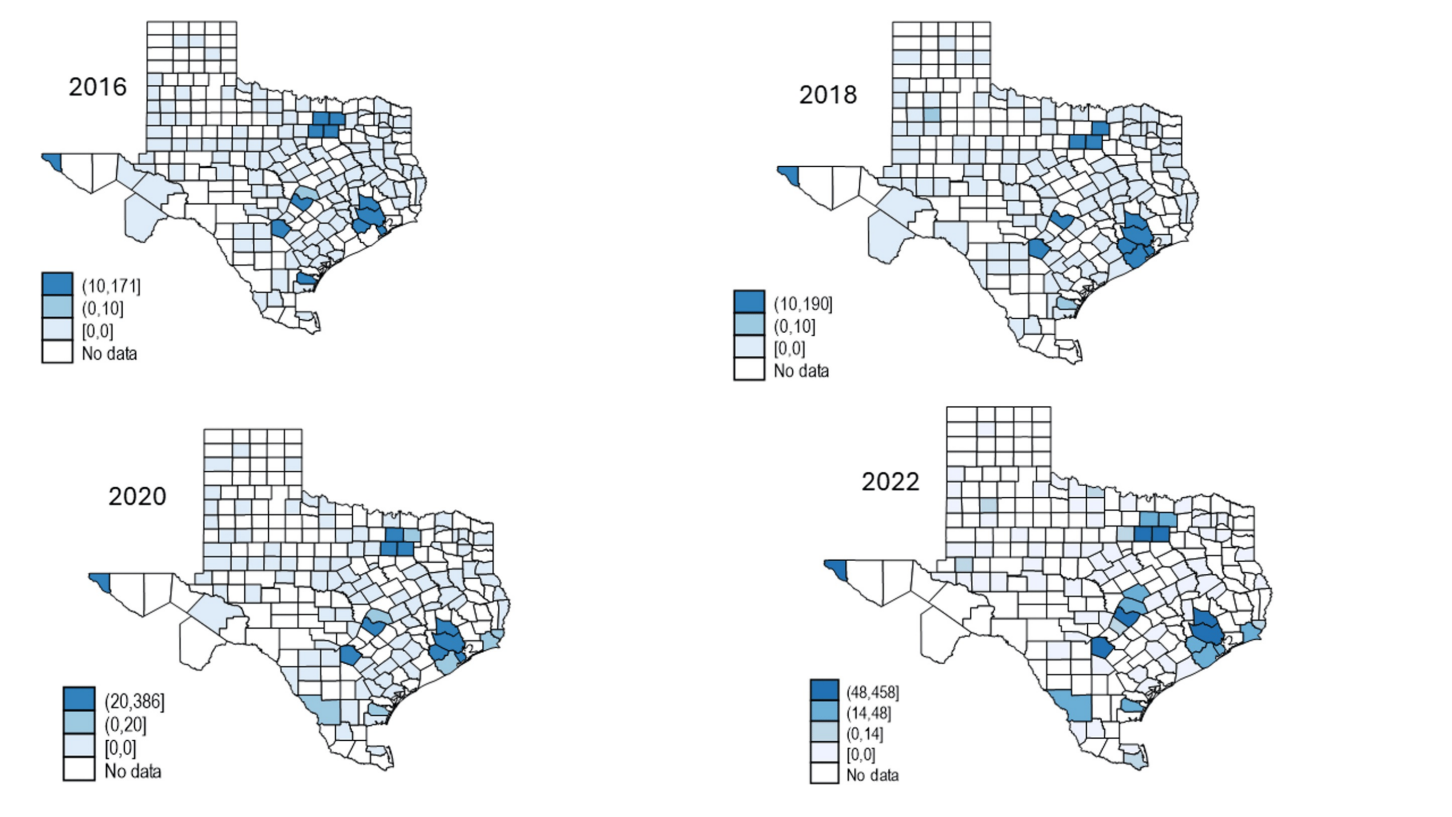
Polysubstance-Related Deaths by County and Year The image was created by the authors of this article using the STATA v17 software program.

Figure 1 illustrates the geographic distribution of drug-related deaths by county and year, and Figure 2 illustrates the geographic distribution of polysubstance-related deaths. The darker the color, the higher the number of deaths. Similar to the finding in Table 1, the number of counties reporting deaths has increased over time. And while the number of deaths have remained steady in big cities like Dallas and Houston, the number is growing in suburb and rural areas.

## Discussion

Trends in death

Our analysis reveals a concerning trend in Texas, with a steady increase in both drug-related and polysubstance-related deaths from 2016 to 2022. The growing number of counties reporting drug-related deaths and the geographical spread out from urban centers into suburban and rural counties suggests that the issue is not confined to specific areas but is a statewide concern. When “place” is considered as the crossing in time and space of various forces such as population change, history, medical resources, and social capital, the growing number of drug-related and polysubstance-related deaths in rural Texas indicates vulnerability and inequity. The rapid escalation in drug-related and polysubstance-related deaths indicates a growing public health crisis.

The data also points to a significant surge in polysubstance-related deaths between 2019 and 2020, coinciding with the onset of the COVID-19 pandemic. This could suggest that the pandemic has exacerbated the issue, possibly due to factors such as increased stress, isolation, and disruption of support services. However, further research would be needed to definitively establish this link and to explore the underlying causes of the overall upward trend in drug-related and polysubstance-related deaths. The findings underscore the urgent need for targeted interventions and strategies to address this escalating crisis.

Potential contributors to drug-related death trends

Access to mental health and substance use treatment programs remains challenging as Texas, a Medicaid non-expansion state, still ranks among the lowest states for access to mental health care [12, 13]. This has been a major constraint for low-income and marginalized individuals who are dependent on government-run programs for mental health. Research shows that states that do not expand programs such as Medicaid have higher rates of uninsured individuals with mental health needs [14, 15]. Expanding access to mental health care improved access to substance use disorder (SUD) treatment programs and decreased the risk of suicide [16, 17].

Texas is also a border state where many assert it serves as a major port of entry for the illegal fentanyl that is fueling the opioid epidemic [18]. The US-Mexico border is a crucial locus in the examination of opioid overdose dynamics due to its complex interplay of socioeconomic and political factors. Conclusions are difficult to draw from current data sets as the U.S-Mexico border is not a homogenous entity, with wide variations not only in culture and population but also in drug trafficking patterns and the cost of illicit drugs [19]. One study demonstrated an overall lower rate of unintentional drug overdose death for New Mexico border residents compared to non-border residents, with a greater reduction in risk of death in the Hispanic population [20]. Data from 2008 to 2017 examining alcohol and drug-related mortality at all border states found a higher total mortality in off-border counties [4]. A comparison of prescription drug misuse between Laredo and Brownsville/McAllen, two socioeconomically and geographically similar border cities in Texas, found a higher prevalence of misuse in Laredo [21]. This data emphasizes the heterogeneity of drug abuse along the US-Mexico border.

Reporting of drug-related deaths in Texas

Data analysis of opioid-associated death in Texas is complicated by the inconsistencies in the state’s reporting system. Currently, the National Vital Statistics System (NVSS) receives and analyses data from death certificates, which include cause-of-death information reported by medical examiners and coroners. The accuracy of the data is contingent upon the information provided, and suspicion of drug toxicity is a prerequisite for involving the local medical examiner or coroner [22]. The entity charged with conducting the autopsy further contributes to the variability in data. Medical examiners are board-certified physicians, while coroners in the State of Texas are often elected officials such as Justices of the Peace with limited medical knowledge [23]. Among Texas’s 254 counties, only 13-15 counties have a medical examiner. Prior to 2019, only counties with populations exceeding one million were mandated to establish medical examiner offices, a threshold that has been increased to two million residents [24]. As of now, only four Texas counties meet these criteria, exacerbating the dearth of vital data and impeding targeted intervention efforts. Funding distribution has been equally uneven. In 2022, Texas received $1,199,006 in overdose prevention funding, with $854,506 going to Harris County alone [25].

Impact of legislation

Furthermore, Texas’ legislative landscape presents formidable obstacles to opioid overdose prevention. Syringe service programs remain illegal in Texas despite data showing that users of syringe exchange services prevent infectious disease transmission and increase the likelihood of entering treatment [26]. The recent prohibition of fentanyl testing strips and weakened “Good Samaritan” laws further deter individuals from seeking help during overdose emergencies, perpetuating preventable fatalities.

Strengths

The paper addresses the ongoing opioid epidemic, a critical public health issue, by providing valuable insights into trends in drug and polysubstance-related deaths in Texas. Utilizing data from the Texas Department of State Health Services’ surveillance system, the study covers a significant period from 2016 to 2022, enabling the identification of long-term trends and patterns. By comparing different counties within Texas, the paper highlights regional disparities and the spread of the opioid crisis from urban to rural areas, emphasizing the need for targeted interventions. Additionally, the paper identifies a significant surge in polysubstance-related deaths during the COVID-19 pandemic, offering timely insights into how the pandemic has exacerbated existing public health challenges.

Limitations

While it presents important information on recent trends in deaths related to drug overdose in the State of Texas, this study has several limitations. The data is drawn solely from Texas Health Data-Opioids, and the results may underestimate the number of deaths given the current challenge of capturing all the drug-related deaths in the state. The assumption that counties without data had no drug-related deaths could lead to inaccuracies. The causes of death were identified through specific ICD-10 codes. Potential errors in coding or different coding protocols for other drug-related deaths would not be included in the data. Similarly, polysubstance-related deaths were identified through specific drug codes. Any misclassification or omission could affect the accuracy of the results. The study also implies a connection between the COVID-19 pandemic and an increase in polysubstance-related deaths. However, without further analysis or data, this remains a correlation rather than a proven causation. The study is limited to Texas and may not be generalizable to other regions or states. The study covers only a specific period (2016-2022). Changes in drug use patterns or reporting practices before or after this period would not be captured. This underscores the need for further studies using multiple data sources to confirm and expand on these findings.

## Conclusions

The current opioid epidemic is a significant national public health concern that calls for a comprehensive and strategic response. Addressing this crisis in Texas necessitates a multifaceted approach, including improved data collection, appropriate resource distribution, and legislative reforms driven by evidence-based data. Enhanced data collection and sharing between counties is the first critical step, providing a clear understanding of the scope and nuances of the crisis, identifying high-risk groups and regions, and tracking the impact of interventions. The second aspect, appropriate resource distribution, ensures that resources such as funding, medical personnel, and treatment facilities are equitably allocated, making care accessible to all affected individuals, including the uninsured. The third component, legislative reforms, involves curbing the trafficking of narcotics across the southern border, enhancing regulations to control opioid prescriptions, promoting alternative pain management methods, and improving access to recovery services. Importantly, these reforms should be guided by solid, empirical data to ensure their relevance and effectiveness. This comprehensive strategy, which combines data-driven decision-making, resource optimization, and evidence-based policy changes, is essential for tackling the opioid epidemic in Texas. This approach not only addresses the immediate crisis but also contributes to the broader discourse on public health policy and practice.
